# Early surgery for superficial supratentorial spontaneous intracerebral hemorrhage: a Finnish Intensive Care Consortium study

**DOI:** 10.1007/s00701-020-04470-y

**Published:** 2020-06-29

**Authors:** Teemu Luostarinen, Jarno Satopää, Markus B Skrifvars, Matti Reinikainen, Stepani Bendel, Sami Curtze, Gerli Sibolt, Nicolas Martinez-Majander, Rahul Raj

**Affiliations:** 1 Division of Anesthesiology, Department of Anesthesiology, Intensive Care, and Pain Medicine, Helsinki University Hospital and University of Helsinki, Topeliuksenkatu 5, PO BOX 266, 00029 HUS Helsinki, Finland; 2 Department of Neurosurgery, Helsinki University Hospital and University of Helsinki, Helsinki, Finland; 3 Department of Emergency Care and Services, Helsinki University Hospital and University of Helsinki, Helsinki, Finland; 4 Department of Anesthesiology and Intensive Care, University of Eastern Finland and Kuopio University Hospital, Kuopio, Finland; 5 Department of Neurology, Helsinki University Hospital and University of Helsinki, Helsinki, Finland

**Keywords:** Intracerebral hemorrhage, Stroke, Surgery, Neurosurgery, Outcome, Mortality

## Abstract

**Background:**

The benefits of early surgery in cases of superficial supratentorial spontaneous intracerebral hemorrhage (ICH) are unclear. This study aimed to assess the association between early ICH surgery and outcome, as well as the cost-effectiveness of early ICH surgery.

**Methods:**

We conducted a retrospective, register-based multicenter study that included all patients who had been treated for supratentorial spontaneous ICH in four tertiary intensive care units in Finland between 2003 and 2013. To be included, patients needed to have experienced supratentorial ICHs that were 10–100 cm^3^ and located within 10 mm of the cortex. We used a multivariable analysis, adjusting for the severity of the illness and the probability of surgical treatment, to assess the independent association between early ICH surgery (≤ 1 day), 12-month mortality rates, and the probability of survival without permanent disability. In addition, we assessed the cost-effectiveness of ICH surgery by examining the effective cost per 1-year survivor (ECPS) and per independent survivor (ECPIS).

**Results:**

Of 254 patients, 27% were in the early surgery group. Overall 12-month mortality was 39%, while 29% survived without a permanent disability. According to our multivariable analysis, early ICH surgery was associated with lower 12-month mortality rates (odds ratio [OR] 0.22, 95% confidence intervals [CI] 0.10–0.51), but not with a higher probability of survival without permanent disability (OR 1.23, 95% CI 0.59–2.56). For the early surgical group, the ECPS and ECPIS were €111,409 and €334,227, respectively. For the non-surgical cohort, the ECPS and ECPIS were €76,074 and €141,471, respectively.

**Conclusions:**

Early surgery for superficial ICH is associated with a lower 12-month mortality risk but not with a higher probability of survival without a permanent disability. Further, costs were higher and cost-effectiveness was, thus, worse for the early surgical cohort.

**Electronic supplementary material:**

The online version of this article (10.1007/s00701-020-04470-y) contains supplementary material, which is available to authorized users.

## Introduction

Spontaneous intracerebral hemorrhage (ICH) is associated with high mortality and morbidity rates [15]. It is a great burden on healthcare systems, and the associated treatment costs are high because it requires an extended rehabilitation period [17, 20]. Despite there being multiple randomized studies into the issue, it is still unclear what the benefits are to surgery being carried out during the early stages of ICH treatment [13, 14, 23].

Using data on ICH patients that were available in a large, retrospective multicenter intensive care unit (ICU) database, we assessed the association between early ICH surgery and mortality rates after spontaneous supratentorial ICH, using the same inclusion criteria that were used for STICH II [14]. We hypothesized that when compared to non-surgical treatment, early surgery would be associated with lower mortality rates but not with an increased rate of survival without permanent disability. A secondary aim was to assess the cost-effectiveness of surgical treatment for ICH. Our hypothesis was that early ICH surgery would be associated with lower cost-effectiveness per 1-year survivor but with increased cost-effectiveness per independent survivor.

## Methods

### Patient selection and variables

We conducted a retrospective, multicenter study using a high-quality ICU database from the Finnish Intensive Care Consortium (FICC). FICC includes prospectively collected data from most of the ICUs in Finland, with the exception of some specialized units. FICC has previously been described in detail [18]. Data on patients in FICC were identified based on their ICU admission diagnosis, after which the diagnoses were manually verified. We included adult patients (≥ 18 years) who had been treated between January 2003 and December 2013 for a spontaneous ICH in any of four tertiary ICUs in Finland (providing neurointensive and neurosurgical care). In Finland, there are five university hospitals that provide neurointensive and neurosurgical care, of which four provided data to FICC during the study period. We only included patients with supratentorial ICHs that were located ≤ 10 mm from the cortex and were between 10 and 100 cm^3^ in size [14]. We excluded readmitted patients and patients with missing Glasgow Coma Scale (GCS) scores.

Three authors who had been blinded to information about the patients’ treatments or outcomes evaluated all of the patients’ computed tomography (CT) scans [3]. Hematoma volume was measured using the ABC/2 method [9]. Midline shift was measured in millimeters. Intraventricular hemorrhage (IVH) in any ventricle was noted. Depth from the cortex was measured in millimeters, from the point closest to the cortex.

Patients in the medical treatment group were treated according to general neurointensive care principles and local guidelines, which are based on the ESO and AHA guidelines [6, 19].

### Outcome and cost variables

Our primary outcome was 12-month mortality rates. The dates of death for all patients were obtained from Statistics Finland. We also report ICU, hospital, and 30-day mortality rates. As a secondary outcome, we used a surrogate variable of survival without permanent disability, with a permanent disability defined as a disability for which the patient was granted a permanent disability allowance or disability pension by the Social Insurance Institute (Kela) in Finland. Kela is a Finnish government agency that is funded by taxes, insurance payments, and municipalities and that provides all social security payments for the country. In order to qualify for a permanent disability allowance or disability pension, Kela mandates that a person must be unable to independently carry out daily activities (e.g., self-hygiene, basic housekeeping, taking care of things outside the home) or to be unable to return to work for a minimum of one consecutive year. We therefore defined all patients who were alive 1 year after ICU admission and who had been granted a permanent disability allowance or pension by Kela (by September 30, 2016) as permanently disabled [16].

The total healthcare cost variable included the index university hospital treatment costs, rehabilitation hospital costs, and social security costs up to 1 year after admission. The cost variables have previously been described in detail [2, 16, 17]. We adjusted all costs according to the average annual Consumer Price Index in Finland into euros, based on the exchange rate in 2019 (data from Statistics Finland):

$$ \mathrm{CPI}\ \mathrm{adjusted}\ \mathrm{costs}=\mathrm{Costs}\times \frac{\mathrm{CPI}\ \mathrm{in}\ 2019}{\mathrm{Admission}\ \mathrm{year}\ \mathrm{CPI}} $$

### Statistical analyses

We used SPSS Statistics 24.0 for Mac (IBM Corp, Armonk, NY) and Stata Statistical Software for Mac (StataCorp LP, College Station, TX) to carry out statistical analyses. We used a *χ*^2^ test to compare categorical data (and a Bonferroni correction when appropriate) and presented the results as percentages (%). We tested continuous data for normality and found that all continuous variables were highly skewed. Thus, we used non-parametric Mann-Whitney *U* and Kruskal-Wallis tests for continuous data between groups. Continuous data are presented as medians with interquartile ranges. We considered *p* values < 0.05 as statistically significant.

To assess the independent association between early ICH surgery and outcomes, we used multivariable logistic regression modeling, adjusting for the severity of illness and likelihood of surgical treatment (primary analysis). We adjusted for severity of illness by creating a severity of illness model that included variables that significantly associated with 12-month mortality in univariate analysis, as well as prognostic factors from our recent study [3]. Thus, the final severity of illness model included the following: age (continuous), GCS score (defined as the worst score during the first 24 h in the ICU or the last score preceding sedation for intubated/sedated patients, in accordance with the Simplified Acute Physiology Score II (SAPS II) definition [4]), a modified SAPS II score (not including the following: age, presence of chronic comorbidity, GCS score), presence of a significant comorbidity (defined according to SAPS II and the Acute Physiology and Chronic Health Evaluation II [8] criteria), ICH volume in cubic centimeters, midline shift in millimeters, and the presence of IVH.

To adjust for differences in the probability of surgical treatment, we created a propensity score, predicting the likelihood of surgical treatment (propensity score regression adjustment [1]). The propensity score regression model accounts for the patient’s age, the hospital, the presence of a significant comorbidity, the ICH location (superficial or deep), the ICH volume (in cubic centimeters), the midline shift in millimeters, and the presence of IVH.

We assessed the performance of the severity of illness model and the propensity score model by calculating their area under the receiver operating characteristic curve (AUC).

We categorized patients into one early ICH surgery cohort and one non-surgical cohort. We defined early ICH surgery as ICH evacuation within 1 day of hospital admission (≤ 1 day). In the primary analysis, patients operated on after the first day (> 1 day) of admission were included in the non-surgical cohort. In a secondary analysis, we included all surgically treated patients (early surgery and late surgery) as one group. To minimize the possibility of early mortality rates affecting the results, we conducted a sensitivity analysis, excluding those dying within 3 days (≤ 3 days).

To evaluate cost-effectiveness, we calculated the effective cost per survivor (ECPS) and effective cost per independent survivor (ECPIS). The ECPS and ECPIS are defined as the sum of costs for all patients divided by the number of 1-year survivors or independent 1-year survivors, respectively (e.g., if the sum of costs for 100 patients is €1,000,000 and there are 20 survivors and 10 independent survivors, the ECPS would be €50,000 and the ECPIS would be €100,000). We calculated the ECPS and ECPIS separately for the early surgical and non-surgical cohorts.

The ethics committee of Helsinki University Hospital (HUS 194/13/03/02/2014) and the Finnish National Institute for Health and Welfare (Dnro THL/1298/5.05.00/2019) approved of this registry study and waived the need for informed consent. The research committees of all participating university hospitals approved the study and the data collection. We conducted the study according to the Strengthening the Reporting of Observational Studies in Epidemiology Guidelines (Online Supplement 1) [14].

## Results

### Baseline characteristics

**Fig. 1:**
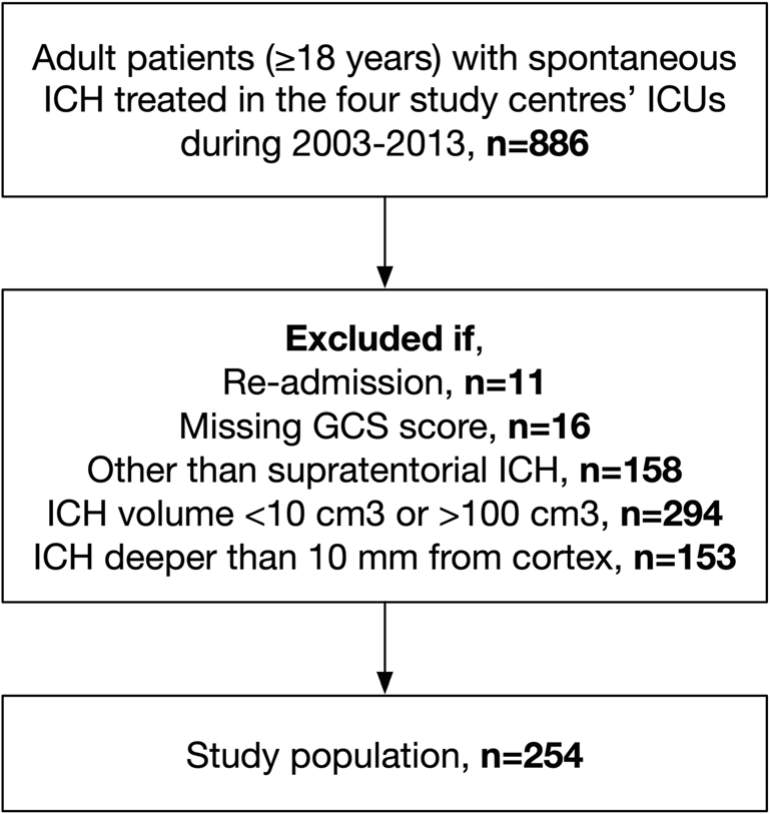
Flowchart of patient inclusion for this study

A total of 254 patients were included (Fig. 1). Of these, 68 patients (27%) were in the early ICH surgery cohort and 186 patients (73%) were in the non-surgical cohort. In the non-surgical cohort, nine patients underwent later ICH surgery (at a median of 3 days, with a range of 2–17 days). Among all patients, 71% were admitted to the ICU from the emergency department, 9% from the ward, 6% from an intermediate care unit, 5% from the operating theater, and 9% from other locations.

Patient baseline characteristics are displayed in Table 1. There were no differences in age, sex, presence of severe comorbidity, pre-admission functional status, or median admission year between the early surgical and non-surgical cohorts. Worst 24-h GCS scores for patients in the early surgical cohort were lower than for patients in the non-surgical cohort (a median GCS score of 8 versus 11). Patients in the early surgical cohort had larger ICH volumes (a median of 31 cm^3^ versus 22 cm^3^). However, there were no differences in the degree of midline shift, depth from the cortex, or presence of IVH. There was no difference in the modified SAPS II score between the groups. Patients in the early surgical cohort had higher levels of treatment intensity. The median ICU stay was slightly longer for those in the early surgical cohort (3 versus 2 days), but hospital length of stay was the same (a median of 6 days).

**Table 1:** Differences in patient characteristics between the surgical cohort and the non-surgical cohort

	All patients (*N* = 254)	Early surgical cohort (*n* = 68)	Non-surgical cohort (*n* = 186)	*p* value
Clinical variables				
Age (years), median (IQR)	62 (53, 71)	65 (58, 71)	61 (51, 71)	0.15
< 45	26 (10%)	4 (6%)	22 (12%)	0.21
45–75	202 (80%)	59 (87%)	143 (77%)	
> 75	26 (10%)	5 (7%)	21 (11%)	
Sex				
Female	97 (38%)	23 (34%)	74 (40%)	0.39
Male	157 (62%)	45 (66%)	112 (60%)	
GCS score, median (IQR)	10 (5, 14)	8 (5, 11)	11 (5, 14)	0.005
13–15	91 (36%)	10 (15%)	81 (44%)	< 0.001
9–12	53 (21%)	21 (31%)	32 (17%)	
3–8	110 (43%)	37 (54%)	73 (39%)	
Severe chronic comorbidity	28 (11%)	6 (9%)	22 (12%)	0.50
Pre-admission antithrombotic medication	22 (9%)	9 (13%)	13 (7%)	0.12
Pre-admission functional ability				
Independent	219 (88%)	57 (88%)	162 (88%)	0.86
Dependent	29 (12%)	8 (12%)	21 (12%)	
Admission year	2009 (2007, 2011)	2009 (2007, 2011)	2010 (2007, 2011)	0.47
Radiological variables				
ICH volume (cm^3^), median (IQR)	25 (16, 39)	31 (23, 47)	22 (15, 37)	0.002
10–29 cm^3^	157 (62%)	32 (47%)	125 (76%)	0.008
30–49 cm^3^	59 (23%)	24 (35%)	35 (20%)	
50–100 cm^3^	38 (15%)	12 (18%)	26 (14%)	
Depth from cortex (mm), median (IQR)	0 (0, 3)	0 (0, 2)	0 (0, 3)	0.18
Midline shift (mm), median (IQR)	4 (0, 9)	6 (0, 9)	3 (0, 9)	0.10
≥5 mm	124 (49%)	40 (59%)	84 (45%)	0.054
Intraventricular hemorrhage	97 (38%)	25 (37%)	72 (39%)	0.78
ICU variables				
ICP monitoring	28 (11%)	12 (18%)	16 (9%)	0.042
External ventricular drain	18 (7%)	6 (9%)	12 (7%)	0.51
Modified SAPS II score*, median (IQR)	14 (8, 20)	17 (8, 22)	14 (8, 20)	0.30
SAPS II score, median (IQR)	35 (24, 51)	40 (28, 54)	33 (23, 50)	0.029
TISS-76 mean score per day, median (IQR)	26 (20, 32)	29 (24, 33)	23 (17, 31)	< 0.001
TISS-76 total score for ICU period, median (IQR)	63 (39, 118)	101 (57, 151)	55 (34, 101)	< 0.001
Duration of stay				
ICU (days), median (IQR)	2 (1, 3)	3 (1, 4)	2 (1, 3)	< 0.001
Hospital (days), median (IQR)	6 (3, 11)	6 (3, 9)	6 (2, 12)	0.72
Outcome				
ICU mortality	28 (11%)	0 (0%)	28 (15%)	0.001
Hospital mortality	47 (19%)	4 (6%)	43 (23%)	0.002
30-day mortality	76 (30%)	15 (22%)	61 (33%)	0.10
12-month mortality	100 (39%)	20 (29%)	80 (43%)	0.050
Alive without a permanent disability	73 (29%)	16 (24%)	57 (31%)	0.27

*Abbreviations*: *GCS* Glasgow Coma Scale, *ICP* intracranial pressure, *ICU* intensive care unit, *IQR* interquartile range, *NA* not applicable, *SAPS II* Simplified Acute Physiology Score II, *TISS* Therapeutic Intervention Scoring System

*SAPS II score without age, GCS, or chronic comorbidities

In univariate analysis, the early surgical cohort had lower ICU and hospital mortality rates than the non-surgical cohort (an ICU mortality rate of 0% versus 15% and a hospital mortality rate of 6% versus 23%). There were no statistically significant differences in 30-day or 12-month mortality rates between the groups (which were, overall, 30% and 39%, respectively). There was no statistically significant difference in the proportion of patients who survived without permanent disability between the groups (which was 29% overall).

### Multivariable analysis

The severity of illness model performed well in predicting 12-month mortality rates (AUC 0.83, 95% CI 0.78–0.88). The severity of illness model performed adequately in predicting the probability of survival without permanent disability (AUC 0.77, 95% CI 0.71–0.84). The propensity score regression model for predicting early surgery yielded an AUC of 0.67 (95% CI 0.59–0.74).

**Fig. 2:**
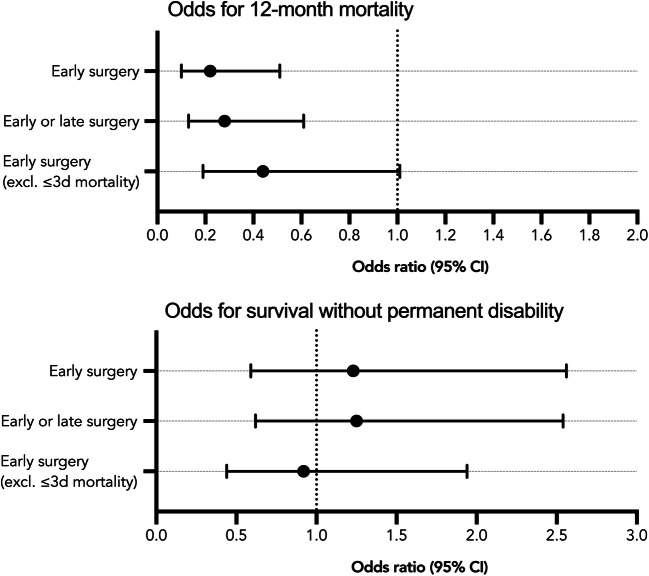
Association between surgery and outcome in patients with spontaneous intracranial hemorrhage. An odds ratio lower than 1 indicates a lower risk of death; an odds ratio more than 1 indicates an increased risk of death

In our primary analysis, when comparing early ICH surgery to non-surgical treatment, early surgery was associated with a lower 12-month mortality rate (OR 0.22, 95% CI 0.10–0.51, *p* < 0.001) (Fig. 2). There was no association between early ICH surgery and the probability of survival without permanent disability (OR 1.23, 95% CI 0.59–2.56, *p* = 0.577).

In the secondary analysis, early or late ICH surgery was associated with a lower 12-month mortality rate (OR 0.28, 95% CI 0.13–0.61, *p* = 0.001), but not with the probability of survival without permanent disability (OR 1.25, 95% CI 0.62–2.54, *p* = 0.527).

In the sensitivity analysis, after excluding patients who died within 3 days of admission (*n* = 43), the association between early ICH surgery and 12-month mortality rates did not reach statistical significance (OR 0.44, 95% CI 0.19–1.01, *p* = 0.053). Further, the sensitivity analysis showed no association between early ICH surgery and the probability of survival without permanent disability (OR 0.92, 95% CI 0.44–1.94, *p* = 0.830).

### Cost-effectiveness

Total 1-year healthcare costs for the early surgical cohort (*n* = 68) were €5,347,630 and, for the non-surgical cohort (*n* = 186), €8,063,873. There were 48 1-year survivors in the early surgical cohort and 106 1-year survivors in the non-surgical cohort. Thus, the ECPS for the early surgical cohort was €111,409 (95% CI €93,551–€129,267), and for the non-surgical cohort, it was €76,074 (95% CI €70,081–€82,068). There were 16 patients and 57 patients who survived without permanent disability in the early ICH surgery group and the non-surgical group, respectively. The ECPIS is therefore €334,227 (95% CI €316,369–€352,085) for the early surgical cohort and €141,471 (95% CI €135,478–147,465) for the non-surgical cohort.

## Discussion

### Key findings

In this retrospective, register-based multicenter study of early surgery versus medical treatment in patients with superficial, supratentorial spontaneous ICH, we found that early surgery was associated with lower 12-month mortality rates when compared to non-surgical treatment outcomes. Our results showed that patients undergoing early ICH surgery appeared to be in worse clinical condition than patients who received only medical treatment, but that after adjusting for disease severity and the probability of receiving surgical treatment, early surgical treatment was associated with lower mortality rates. However, early surgical treatment was not associated with an increased probability of survival without permanent disability. Further, surgical treatments led to higher treatment-related costs and were less cost-effective than conservative treatments (resulting in higher ECPS and ECPIS).

### Comparison with previous studies

The role of surgery in the treatment of spontaneous ICH has been a topic of debate for decades. Despite multiple studies assessing the benefits of ICH surgery, it has remained a controversial treatment option [10, 13, 14, 23]. One potential reason for this uncertainty is the heterogeneity of the disease. The location, size, and symptoms of ICH are highly variable, and there is a wide spectrum of differences in patients’ medical histories. In clinical practice, one of the main determinants when considering ICH surgery is the location of the hemorrhage. Deep-seated clots are difficult to reach, whereas patients with subcortical hemorrhages are more often offered surgical treatment. Reducing mass effect and alleviating consequent secondary neural damage are considered the main benefits of ICH surgery; removal of the clot may also attenuate the potentially toxic effects that blood byproducts can have and prevent the formation of edema in the surrounding brain tissue [12, 22]. Interestingly, our propensity score for predicting the probability of early ICH surgery displayed an AUC of only 0.67, indicating that the indications for ICH surgery seem to vary in a pseudorandomized manner.

A meta-analysis of 14 randomized controlled trials (RCT) investigating the role of surgery in spontaneous ICHs concluded cautiously that early surgery before deterioration in clinical condition might be beneficial, bearing in mind that this was a very heterogenous group of studies in terms of design, information used, and outcomes [5]. For example, the distance between the hematoma and the cortex was mentioned only in one study. The STICH trial was the first large international, multicenter RCT on early surgery versus best medical treatment in patients with supratentorial spontaneous ICH [13]. The results were neutral, since surgery for superficial lesions improved outcomes while surgery for deeper lesions had the opposite effect. This finding led to another RCT, the STICH II trial, in which the authors selected patients who had benefitted from surgery to be randomized into ICH surgery or best medical treatment [14]. These patients had superficial (≤ 1 cm) supratentorial ICHs with a volume between 10 and 100 ml (the same as in the present study). STICH II eventually concluded that there was no benefit to early surgery compared to conservative treatment; however, 21% of the patients initially assigned to the best medical treatment group were operated on later during the study period because of clinical deterioration. Thus, although intention-to-treat analyses were used, the high rate of crossover from the medical to the surgical group may have affected the results.

In this study, we used similar inclusion criteria to STICH II as a means of assessing the potential benefits of surgery in real-life clinical situations. In STICH II, the authors tried to select patients that presented a true clinical equipoise for the attending physicians, whereas our data consist of the results of clinical case-by-case decision-making [14]. It is also worth noting that STICH II defined early surgery as surgery within 12 h of ictus, whereas we defined it as within 24 h of admission. Moreover, we did not exclude patients with IVH. All in all, our results were somewhat encouraging, showing a clear association between early ICH surgery and lower 12-month mortality rates in the surgical group versus the non-surgical group. Nevertheless, we found no association between early surgery and the probability of survival without permanent disability, suggesting that ICH surgery may help avoid mortality, but does not improve neurological outcomes. In addition, we performed a secondary analysis that included patients who required delayed surgery. In this secondary analysis, ICH surgery was associated with a reduced 12-month mortality rate when compared to non-surgical treatment. Another recent retrospective study from Finland also found that ICH surgery was associated with lower mortality rates, especially among patients under 70 years old, despite large hematoma volumes. [10]

An interesting finding in our study was that despite patients in the non-surgical group having higher 24-h GCS scores (i.e., being in better clinical condition) significantly lower clot volumes, their in-hospital mortality rate was still significantly higher than that of the early surgery group. Although the baseline variables did not show significant differences between these patient groups, there may still be some hidden confounding factors—especially since this is a retrospective registry study—that led these patients to receive only medical treatment. However, the positive effects of ICH surgery itself may also play a role in explaining this difference.

It is well known that spontaneous ICH is one of the main burdens on healthcare services, incurring high costs partly because patients often need long-term rehabilitation [7]. Compared to subarachnoid hemorrhages (SAH), traumatic brain injuries (TBI), and acute ischemic stroke, spontaneous ICH is associated with the highest rate of permanent disability and ECPS [17]. Our results showed higher treatment costs for the early surgery group. This may be at least partly due to longer ICU stays and additional costs related to the surgery itself, but it could also be related to the fact that these patients survive and, thus, require prolonged and intensive rehabilitation. It is a self-fulfilling prophecy if clinicians withhold the most aggressive surgical interventions, which may save lives, to severely ill ICH patients, that it will be financially cheaper but possibly unethical. Thus, in assessing healthcare costs, it is preferable to use measures of cost-effectiveness. The most commonly used measure of cost-effectiveness is the cost of a quality adjusted life year (QALY) [21]. Cost-effective treatments are defined as having a QALY of approximately €25,000–€35,000 [11]. Without data on quality of life, cost-effectiveness may be quantified as ECPS or, more appropriately, ECPIS. There are no limits to or recommendations for cost-effective treatments in terms of ECPS or ECPIS, and these measures cannot be directly compared to QALY. Still, compared to severe TBI (with an ECPS of approximately €80,000 and an ECPIS of approximately €145,000 [16, 17]), SAH (an ECPS of approximately €70,000, an ECPIS of approximately €95,000 [17]), acute ischemic stroke (an ECPS of approximately €55,000, an ECPIS of approximately €105,000 [17]), and cardiac arrest (an ECPS of approximately €95,000, an ECPIS of approximately €100,000 [2]), ICH surgery seems to be cost-effective in terms of survival, with an ECPS of approximately €110,000, though not in terms of independent living (with an ECPIS of approximately €335,000).

### Strengths and limitations

By using a high-quality, multicenter ICU database like FICC, we were able to collect and analyze prospectively collected data for all patients treated consecutively in participating ICUs. FICC has been extensively validated, and all cases included in the study were also reviewed to confirm diagnoses. In addition, follow-up was comprehensive, and due to the unique landscape of the healthcare system in Finland, we were able to collect full cost-related data for all patients.

Nonetheless, there are some limitations to be considered. First, given the retrospective nature of the study, we can only report associations. Second, we were unable to control for treatment restrictions and withdrawal of care. Ignoring withdrawal of care decisions may affect the results, as it leads to a self-fulfilling prophecy due to withholding treatment as a result of an assumed poor prognosis. To account for this potential source of bias, we conducted a separate secondary analysis that included only those patients who were alive 3 days after admission. This analysis showed a lower 12-month mortality rate for the early surgery cohort; however, this did not quite reach statistical significance (possibly due to lack of power). Third, the FICC database only includes patients treated in the ICU and not in stroke units, where most Finnish ICH patients are treated. Our ICH patients therefore represent a specific subset of patients. Fourth, we used the GCS score as defined by the SAPS II criteria, i.e., the worst GCS score observed during the first 24 h or the last score before a patient was sedated. In other words, the GCS scores may have been measured at different points for different patients. Fifth, we used admission CT scans and, thus, did not consider the dynamic nature of ICH. For example, we could not verify that the improvement in survival was due to a smaller post-operative ICH volume in operated patients. Sixth, ICH volumes were measured using the ABC/2 method and not by more modern volumetric methods. Still, this should have minimal effect on our results as the same methods were applied to both groups. Seventh, we used a surrogate marker of permanent disability instead of the more commonly used modified Rankin scale or the Glasgow Outcome Scale. This also means that one should not convert our results directly into these outcome measures.

## Conclusion

Early surgery for superficial, supratentorial spontaneous ICH treated in the ICU may reduce mortality but that it does not affect the probability of survival without permanent disability. However, treatment costs are higher and cost-effectiveness is lower when compared to patients who are treated more conservatively. The role of surgery in spontaneous ICH remains debatable. Further studies are needed in order to identify which ICH patients may benefit from early surgery.

## Electronic supplementary material

ESM 1 (DOCX 31.8 kb)
